# Network Analysis of Mindfulness Facets, Affect, Compassion, and Distress

**DOI:** 10.1007/s12671-020-01555-8

**Published:** 2020-11-26

**Authors:** Oleg N. Medvedev, Matti Cervin, Barbara Barcaccia, Richard J. Siegert, Anja Roemer, Christian U. Krägeloh

**Affiliations:** 1 University of Waikato, School of Psychology, Hillcrest, Hamilton, 3216 New Zealand; 2 Lund University, Lund, Sweden; 3 Skåne Child and Adolescent Psychiatry, Lund, Sweden; 4 Sapienza University of Rome, Rome, Italy; 5 Auckland University of Technology, Auckland, New Zealand

**Keywords:** Mindfulness, Affect, Compassion, Depression, Anxiety, Stress, Network analysis

## Abstract

**Objectives:**

Mindfulness, positive affect, and compassion may protect against psychological distress but there is lack of understanding about the ways in which these factors are linked to mental health. Network analysis is a statistical method used to investigate complex associations among constructs in a single network and is particularly suitable for this purpose. The aim of this study was to explore how mindfulness facets, affect, and compassion were linked to psychological distress using network analysis.

**Methods:**

The sample (*n* = 400) included equal numbers from general and student populations who completed measures of five mindfulness facets, compassion, positive and negative affect, depression, anxiety, and stress. Network analysis was used to explore the direct associations between these variables.

**Results:**

Compassion was directly related to positive affect, which in turn was strongly and inversely related to depression and positively related to the observing and describing facets of mindfulness. The non-judgment facet of mindfulness was strongly and inversely related to negative affect, anxiety, and depression, while non-reactivity and acting with awareness were inversely associated with stress and anxiety, respectively. Strong associations were found between all distress variables.

**Conclusions:**

The present network analysis highlights the strong link between compassion and positive affect and suggests that observing and describing the world through the lens of compassion may enhance resilience to depression. Taking a non-judging and non-reacting stance toward internal experience while acting with awareness may protect against psychological distress. Applicability of these findings can be examined in experimental studies aiming to prevent distress and enhance psychological well-being.

The last few decades have seen increasing dissemination of application of mindfulness in numerous fields, and mindfulness-based interventions (MBIs) have been implemented in order to promote wellness, reduce stress, and treat various mental disorders (Michalak et al. 2020). Especially in times of the current COVID-19 pandemic, where millions of people around the world suffer from the consequences of lockdowns, social distancing, separation from families and friends, and financial and workplace pressure (Kumar and Nayar 2020), a better understanding of factors that protect from psychopathological distress is warranted. Mindfulness refers to the capacity of paying attention to the present moment with awareness and without judging one’s inner experiences such as thoughts, emotions, impulses, and physical sensations (Kabat-Zinn 1990). Individuals can be “mindful” to a greater or lesser extent independently from the practice of formal mindfulness, although the latter trains people to cultivate awareness regularly for prolonged periods of time thus making it possible to achieve an increasingly mindful attitude (Brown and Ryan 2003). Mindfulness is associated with emotional well-being (Bränström et al. 2011; Kong et al. 2014; Lyvers et al. 2014; Malinowski and Lim 2015) and fewer symptoms of depression and anxiety (Cheung and Ng 2019). MBIs have also been proven effective in reducing stress, anxiety, and depression (Khoury et al. 2015; Krägeloh et al. 2019; Williams et al. 2014; Zhou et al. 2020). Furthermore, mindfulness practice has been utilized for the treatment of mental disorders by helping individuals to increase awareness of both internal and external stimuli and repeatedly re-directing their attention (e.g., to the breath) in order not to be overwhelmed by distressing emotions, sensations, and thoughts (Barcaccia and Couyoumdjian 2018; Krägeloh et al. 2019).

With a now well-established evidence base for the benefits of a mindful disposition and mindfulness training, the research literature has extended to explore the relevance of wider contextual factors. One of these factors is the role of compassion, and in the context of Buddhist ideas that inspired the development of MBIs, compassion may be regarded as the intrinsically motivated expression of ethical guidelines (Krägeloh 2016). Criticisms of MBIs of being too focused on individual therapeutic goals that are in contrast with Buddhist teachings of cultivating mindfulness alongside ethics and wisdom (Stanley et al. 2018) have resulted in a variety of responses. On one hand, it has been argued that MBIs provide a short-term approach that establishes the basis for deeper practice that includes compassion, while arguments have also been advanced that ethics and compassion are likely to develop naturally even in the absence of specific instructions (Krägeloh 2016). However, to recognize the trend for a larger variety of mindfulness programs with specific themes and orientations, Singh et al. (2014) introduced the distinction between first- and second-generation MBIs. While the former includes the secular programs such as mindfulness-based stress reduction (Kabat-Zinn 1990), which were designed to address specific psychological issues (e.g., chronic pain), the latter includes more long-term lifestyle interventions as well as MBIs that have a specific focus. The role of compassion for others alongside mindfulness practice is thus more likely to be of relevance in second-generation MBIs.

Apart from MBIs, the importance of compassion has also been acknowledged in various other fields of human activity, ranging from health services (Cochrane et al. 2019), to education (Al-Ghabban 2018), leadership (Shuck et al. 2019), and social policy (Finkel 2019). For example, *Compassion in Practice* is a policy introduced in the UK to promote a culture of compassionate practice in the healthcare system, involving compassionate relationships among doctors, nurses, patients, and health managers (Ling et al. 2020). Nevertheless, very few studies have explored the function of compassion in relation to emotional well-being and its potential role to protect against psychopathology.

Compassion is an innate feature of human nature, characterized by being other-focused, i.e., oriented to improve the well-being of another fellow human (Ling et al. 2020). Compassion has been defined as an “attitude toward other(s), either close others or strangers of all of humanity; containing feelings, cognitions, and behaviors that are focused on caring, concern, tenderness, and an orientation toward supporting, helping, and understanding the other(s), particularly when the other(s) is (are) perceived to be suffering and in need” (Sprecher and Fehr 2005, p. 630). Thus, this dimension implies being aware of suffering experienced by someone, being capable to emotionally resonate with it, understanding the ubiquity of human suffering as part of the human condition, and being willing to alleviate it (Chiesi et al. 2020). Compassion is a distinct concept from empathy and altruism. In fact, when compared to empathy, compassion is a longer lasting state, and thus, it may contribute to lasting prosocial behavior (Sprecher and Fehr 2005). Moreover, compassion implies a behavioral activation, not merely a cognitive and emotional resonance with others’ suffering, as empathy usually entails. Although the concept of altruism is close to that of compassion, the motivations for acting in an altruistic way may be due to a range of reasons, not only compassion (Chiesi et al. 2020).

While it has been widely explored how mindfulness implies a non-judgmental and compassionate attitude toward oneself, it has been less thoroughly investigated how a mindful attitude is related to a non-judgmental and compassionate attitude that is other-directed. In order to address this important topic, it is worthwhile considering the close connection between a non-judgmental self-attitude and self-compassion. According to Neff (2003), a non-judgmental understanding of human vulnerability, both ours and of others, is entailed in self-compassion. In fact, Neff (2003) considers self-directed compassion as a pathway to interpersonal compassion, since it implies the capacity to see one’s own weaknesses and failings in light of the common human experience, knowing that failures and flaws are all part of the human condition. Therefore, every person is worthy of warmth, re-assurance, and compassion, and a compassionate stance toward oneself should also allow for a more compassionate attitude toward our fellow human beings. In addition, in Gilbert’s (2010) definition of compassion, non-judgment has an important role, representing one of its six facets, besides sensitivity, sympathy, empathy, caring, and distress tolerance. In this perspective, non-judgment means being accepting of another person, despite his/her behavior, which may be unpalatable and cause negative emotions such as anger, anxiety, fear, or disgust. Thus, being compassionate toward someone else may mean being non-judgmental and accepting of them, be they friends or strangers, or even enemies, a view that is also shared by Buddhism and Christianity (Barad 2007; Polinska 2007). According to Anālayo (2015), Early Buddhist teachings show that the Buddha never mentioned self-compassion at all, instead he taught only about compassion which is all encompassing. Notwithstanding these considerations, there was a discussion whether self- and other-directed compassion are actually two aspects of the same overarching construct, which warrants future empirical research (Neff and Pommier 2013; Strauss et al. 2016).

Nevertheless, extensive research has incontrovertibly demonstrated the role of self-compassion in well-being, showing that it is negatively related to depression both in the general population and in depressed patients (Krieger et al. 2013; MacBeth and Gumley 2012). For example, Neff (2003), Neff and McGehee (2010), and Raes (2010) found that self-compassion was negatively associated with depression and anxiety. Van Dam et al. (2011), in a large community sample of adults seeking help for depression and/or anxiety, found that self-compassion was a robust predictor of lower depressive and anxious symptomatology, and higher quality of life. In contrast, very little research has been conducted on the role of other-directed compassion (Mongrain et al. 2011) and its role in relation to psychological well-being and psychopathology. A better understanding of this important construct could also shed light on potential interventions aimed at preventing and treating psychological problems and promoting well-being.

Far from being a unitary construct, mindfulness is recognized to be a multi-facet concept (Vago and Silbersweig 2012), characterized by various features. Trait-mindfulness can be assessed through several measures, although the Five Facet Mindfulness Questionnaire (FFMQ; Baer et al. 2006) has the advantage over other instruments of being developed based on the items of several pre-existing mindfulness measures. The FFMQ is the most widely used multi-facet mindfulness scale, entailing five fundamental facets of mindfulness. These facets include the following: observing (identifying and paying attention to internal/external phenomena); describing (using words to describe the observed phenomena); acting with awareness (dedicating oneself totally to the current activity); not judging (having a non-judgmental attitude toward thoughts and emotions); and not reacting (accepting thoughts and emotions by letting them come and go without getting distracted by them) (Baer et al. 2006, 2008).

Recent research has started to focus on the relationship between specific facets of mindfulness and psychopathology symptoms such as depression, anxiety, and stress in non-clinical samples (Medvedev et al. 2018). Particularly, being judgmental toward one’s inner thoughts, feelings, and sensations, and acting with unawareness, is associated with more psychiatric symptoms (Baer et al. 2006). Individuals who tend to adopt a non-judgmental stance toward their own thoughts and feelings tend to have lower depression and anxiety (Cash and Whittingham 2010). Keng and Liew (2017) reported negative associations between the FFMQ total score and both depression and anxiety scores. Royuela-Colomer and Calvete (2016) reported moderate negative correlations between depression and the non-judgment facet, the acting with awareness facet, and the FFMQ total score. Petrocchi and Ottaviani (2016) showed that non-judgment was the only mindfulness facet that inversely predicted depressive symptoms 2 years later. Barcaccia et al. (2019) demonstrated that the non-judgment scale of the FFMQ is not only negatively correlated with maladjustment measures but also inversely predicts depression. Finally, in a recent longitudinal three-wave study, Tumminia et al. (2020) showed that, in a non-clinical sample of adolescents, higher levels of non-judgment predicted longitudinal reductions in rumination that, in turn, predicted longitudinal reductions in negative affect. It seems, therefore, that the non-judgment dimension has a particularly significant role in psychological well-being: the more you judge your internal experience, the worse you feel (Barcaccia et al. 2019).

These observations suggest a complex interplay between positive and negative affect, depression, anxiety, compassion, and the various facets of mindfulness. However, how these variables are interrelated is poorly understood due to methodological limitations in which only a subset of variables have been analyzed in each individual study. Furthermore, these variables have most often been divided into independent (i.e., causes) and dependent variables (outcomes) although they are often theorized to be reciprocally linked. Thus, the investigation of relationships among affect, emotional distress, compassion, and facets of mindfulness would greatly benefit from statistical techniques specifically developed for identifying relationships that may be mutually maintaining or reciprocal. Network analysis is a powerful novel methodology that permits to evaluate the complex relationships among numerous psychological constructs in one principle network (Borsboom and Cramer 2013; Epskamp et al. 2018). The network perspective of psychopathology was recently advocated as the most comprehensive way to outline how key symptoms and risk factor of psychopathology are interrelated (e.g., see Cervin et al. 2020; Mullarkey et al. 2019; Rouquette et al. 2018). A major limitation of earlier network studies was the focus on symptoms of emotional distress while disregarding the importance of potential protective factors, such as mindfulness, compassion, and positive affect and unique links between these factors and psychopathology (Veed et al. 2019). In an exception, Barcaccia et al. (2020) employed network analysis to examine whether a line of protective factors was linked to psychopathology. They found, using a large adolescent sample, that mindfulness and self-reassurance were uniquely related to lower levels of psychopathology. To the best of our knowledge, no study has investigated a complex network relating mindfulness facets, compassion, positive and negative affect, and distress variables such as depression and anxiety. Network analysis is the most appropriate methodology to model unique links between these variables and verify theorized reciprocal associations among them. Furthermore, network analysis provides composite estimates summarizing how important each variable is in the full network (or aspects of the network), which may have clinical implications that extend isolated associations between pre-specified variable pairs.

The aim of the present study was to investigate the direct associations between mindfulness facets, positive affect, and compassion as dimensions of adjustment and depression, anxiety, negative affect and stress as dimensions of maladjustment through network analysis. Variables that play a central role in such a network can be identified based on the literature on centrality (Bringmann et al. 2019; Epskamp et al. 2018) and inform research on prevention and treatment of affective conditions, which is especially important in the current COVID-19 circumstances. An investigation of this complex pattern of interaction could add to our understanding of (1) the possible links between mindfulness facets, affect, and variables of maladjustment such as depression and anxiety and (2) the specific role of compassion for others in relation to mindfulness facets, affect, depression, and anxiety.

## Method

### Participants

This study used data collected from 400 participants in New Zealand including equal numbers from general and student populations. No incentives such as an academic credit or monetary rewards were provided to participants to compensate for their participation in the study. The sample included 93 males (23%); six participants did not indicate their gender. Ages of participants ranged from 17 to 95 years (mean = 38.09; SD = 20.40). The majority of the sample were Caucasian (67%) and the remaining Polynesian (14%), Asian (6%), and others (12%). Parts of this dataset, limited to distress and mindfulness variables only, have been used for psychometric validations (Medvedev et al. 2017, 2020) and regression analyses (Medvedev et al. 2018) published earlier.

### Procedures

Participants from the general population received a survey delivered into their randomly selected mailboxes by the researchers. Surveys were proportionally allocated across five major regions of Auckland, New Zealand. Participants were provided with a self-addressed pre-paid envelope and mailed completed surveys back to the researchers with a response rate of 12%. Student participants completed questionnaires in the lectures during breaks and submitted the survey to the allocated collection box or returned it to the researchers.

### Measures

The 39-item FFMQ (Baer et al. 2006) is a self-report instrument designed in a 5-point Likert scale format to assess the five facets of mindfulness using response options from 1 = “Never or very rarely true” to 5 = “Very often or always true.” The FFMQ subscales comprise act aware (acting with awareness), describing, observing, non-reactivity (non-reactivity to inner experience), and non-judgment (not judging internal experience). There are 19 negatively worded items that need reverse coding prior to data analysis. This measure has been validated in New Zealand using Rasch methodology, and ordinal-to-interval conversion tables were developed and used in the current study to transform ordinal scores into interval-level data (Medvedev et al. 2017).

The Depression, Anxiety, and Stress Scale (DASS-21; Lovibond and Lovibond 1995) is a self-report instrument in a 4-point Likert scale format using response options ranging from 0 = “Did not apply to me at all” to 3 = “Applied to me very much, or most of the time.” DASS-21 is a non-clinical measure of depression, anxiety, and stress. The DASS was recently validated using Rasch analysis and ordinal-to-interval conversion tables for the proposed 20-item scale version of the best fit were published (DASS-20; Medvedev et al. 2020) and used in this study to increase precision of this measure.

The Positive and Negative Affect Scale (PANAS; Watson et al. 1988) is a 20-item self-report scale assessing positive and negative emotions. Participants rate each adjective describing an emotional state on a scale from 1 to 5 (1 = very slightly or not at all and 5 = extremely), reporting their feelings during the previous week. It comprises two subscales, the positive affect (PA) (sample items “interested,” “proud”) and the negative affect (NA) (sample items “distressed,” “jittery”).

The Santa Clara Brief Compassion Scale (SCBCS; Hwang et al. 2008) is a brief, five-item, validated version of the Compassionate Love Scale (Sprecher and Fehr 2005). The five items included in the scale are as follows: (1) “When I hear about someone (a stranger) going through a difficult time, I feel a great deal of compassion for him or her”; (2) “I tend to feel compassion for people, even though I do not know them”; (3) “One of the activities that provides me with the most meaning to my life is helping others in the world when they need help”; (4) “I would rather engage in actions that help others, even though they are strangers, than engage in actions that would help me”; (5) “I often have tender feelings toward people (strangers) when they seem to be in need.” The response scale ranges from 1 (not at all true of me) to 7 (very true of me).

### Data Analyses

Associations between the five facets of mindfulness, compassion, positive and negative affect, depression, anxiety, and stress were explored using a partial correlation network in which unique associations (i.e., pairwise dependencies) between variables were estimated by accounting for all linear relations present in the data. In a partial correlation network, unique associations are termed edges, which are depicted as lines when plotted. Edges connect variables that are called nodes, which are depicted as circles when plotted. Skewness and kurtosis for the variables were reviewed to identify whether transformation was deemed necessary and multivariate normality was tested using R-package *MVN*. If the assumption of multivariate normality was not met, an a priori decision was made to transform the variables using nonparanormal transformation and then analyze whether results appeared to differ. The nonparanormal transformation algorithm transforms a set of observed values to a set of values from a normal distribution. Regularization was used when estimating the networks. This technique shrinks all edges so that spurious edges are set to zero resulting in a sparse final network (Epskamp et al. 2018).

The estimated parameters in the partial correlation network were used to create a plot in which nodes and edges were placed in a two-dimensional space using the force-directed Fruchterman-Reingold algorithm. This algorithm places nodes by taking into account the set of edges present for each node and places nodes with many and strong edges centrally and strongly connected node pairs closely while also avoiding node and edge overlap.

To address the major research question of the present study (i.e., links between protective and maladjustment nodes), we estimated bridge strength centrality. Centrality within a network analytic framework is a measure of how important a node is in the network, that is, the number and strengths of its edges to other nodes. Strength centrality sums all positive and negative edges into a composite centrality score and is the most useful measure in the present study because the inclusion of protective and maladjustment variables is expected to result in both positive and negative edges of which both are important. Bridge strength centrality is a special form of centrality in which only edges that occur between pre-specified groups of nodes are accounted for when estimating centrality. Accordingly, bridge strength centrality makes it possible to examine which nodes are most important in linking protective and maladjustment variables. Bridge strength centrality was estimated using functionality implemented in the R-package *networktools* and we used two pre-specified groups of nodes: protective nodes (mindfulness facets, compassion, and positive affect) and maladjustment nodes (depression, anxiety, stress, and negative affect). Because there were fewer nodes in the maladjustment group of nodes, we used a normalization function that accounts for different number of nodes by averaging bridge strength centrality by the number of possible bridge edges.

The accuracy of the network parameters was explored using bootstrap and case-dropping techniques. Specifically, through 250 bootstraps, a 95% confidence interval around each edge was produced. These bootstrapped confidence intervals were then used to estimate whether two edges were statistically significantly different from each other (Epskamp et al. 2018). Case-dropping was used to examine the robustness of the centrality of each variable within the full network and for the bridge strength centrality estimates. Case-dropping produces a correlation stability (CS) coefficient that denotes the proportion of the full sample that can be dropped from the analysis before violating a correlation of 0.70 between the order of the original centrality estimates and the order of centrality estimates computed using the estimated edges in the case-dropped sample. The highest obtainable CS coefficient is 0.75. CS coefficients above 0.30 are recommended to interpret centrality results, and CS coefficients above 0.50 are considered indicative of accurate centrality estimations (Epskamp et al. 2018).

## Results

Marida’s test indicated multivariate skewness and kurtosis (*p*s < 0.001), but skew and kurtosis for the separate variables were in the adequate range. Table 1 includes descriptive statistics, Cronbach’s alpha (*α*), and skewness and kurtosis values across study variables. As a sensitivity analysis, all variables were transformed using the nonparanormal method described above and we investigated the correlation between the regularized partial correlation matrix produced using transformed variables and the regularized partial correlation matrix produced using non-transformed variables. The correlations between the correlation matrices, using both Pearson’s *r* and Spearman’s rank correlation, were very high (0.99 for both methods). We continued with the non-transformed variables as this let us base the analyses on all data from all participants (i.e., participants with missing data were not excluded). The rate of missing data was very low (0.4%) and missingness was handled using pairwise deletion.

**Table 1:** Descriptive statistics, Cronbach’s alpha (*α*), and skewness and kurtosis values across study variables

Variable	*α*	Mean	SD	Min	Max	Skew	Kurtosis
Compassion (SCBCS)	0.89	25.44	25.44	5.00	35.00	− 0.66	0.34
Act aware (FFMQ)	0.87	24.97	24.97	13.02	40.00	0.50	1.82
Describing (FFMQ)	0.88	23.52	23.52	9.22	35.00	0.29	0.52
Nonjudge (FFMQ)	0.90	25.35	25.35	8.00	40.00	0.14	1.35
Non-react (FFMQ)	0.77	18.33	18.33	11.74	30.00	0.62	0.90
Observing (FFMQ)	0.77	26.08	26.08	16.17	37.08	0.68	0.90
PANAS positive	0.89	34.21	34.21	14.00	49.00	− 0.35	− 0.09
PANAS negative	0.90	19.70	19.70	10.00	45.00	0.88	0.16
Depression (DASS)	0.89	4.39	4.39	0.00	18.00	0.59	− 0.42
Anxiety (DASS)	0.84	5.34	5.34	0.00	21.00	0.53	0.38
Stress (DASS)	0.86	8.22	8.22	0.00	21.00	0.13	0.19

**Fig. 1:**
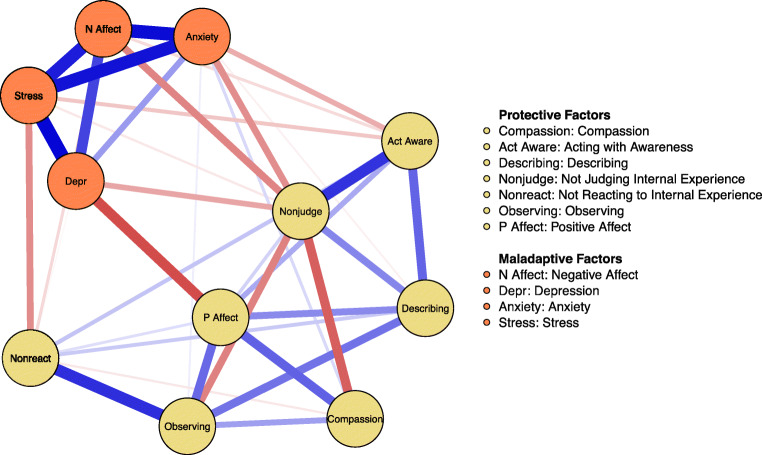
The network structure of the full set of protective and maladaptive nodes. *N*odes (variables) are depicted as circles and unique associations between nodes as lines. Blue lines indicate positive associations. Red lines indicate negative associations. The groups of nodes are indicated using different colors

The network structure of the full set of variables is presented in Fig. 1. The variables depression, stress, and anxiety from the DASS-21 clustered together with the negative affect subscale of the PANAS, thus forming a highly correlated network of maladaptive variables. This is in contrast with the remaining variables that are typically considered to be proteclive factors, namely the five facets of mindfulness, the positive affect subscale of the PANAS, and compassion for others. Within the overall network, nonjudging and positive affect can be considered as bridging variables that present with the strongest inverse association with the maladaptive factors. Of note is that nonjudging was negatively associated with observing and compassions for others.

**Fig. 2:**
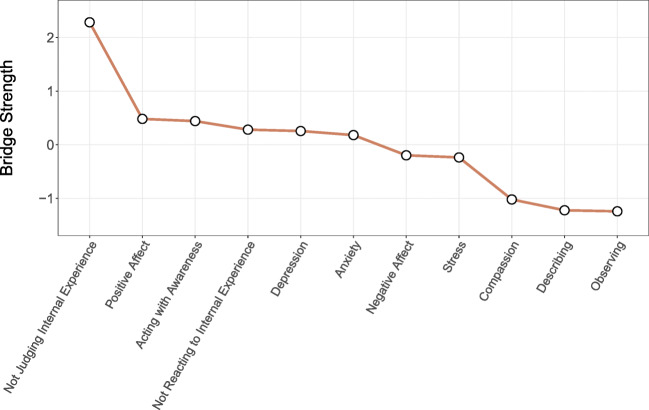
Z-standardized bridge strength centrality for links between protective and maladaptive nodes. *B*ridge strength centrality is an aggregate estimate for the strength of unique associations for a specific node with nodes of a pre-specified group of nodes (a node community). In this study, protective nodes (mindfulness facets, compassion, and positive affect) and maladaptive nodes (depression, anxiety, stress, and negative affect) were used as pre-specified groups of nodes, and bridge strength centrality indicates to what degree each node acts as a bridge between these two group of nodes

To examine nodes importance in linking protective and maladjustment variables, we estimated bridge strength centrality between these two groups of nodes. Results are presented in Fig. 2. Nonjudging internal experience stood out as the node that was most important in linking the two node groups; this node was uniquely and negatively associated with all aspects of maladaptive distress indicating that higher ratings on this variable were uniquely related to lower ratings on all the maladaptive variables. Acting with awareness and not reacting to internal experience (non-reactivity) had markedly higher bridge strength centrality scores compared to compassion, observing, and describing nodes. Of note, the only weak links to maladaptive nodes for compassion and observing were positive edges to anxiety; these were the only two positive edges between the two groups of nodes. The bridge strength centrality for the maladaptive nodes was roughly in the same range for all nodes with a somewhat lower centrality score for the stress node.

**Fig. 3:**
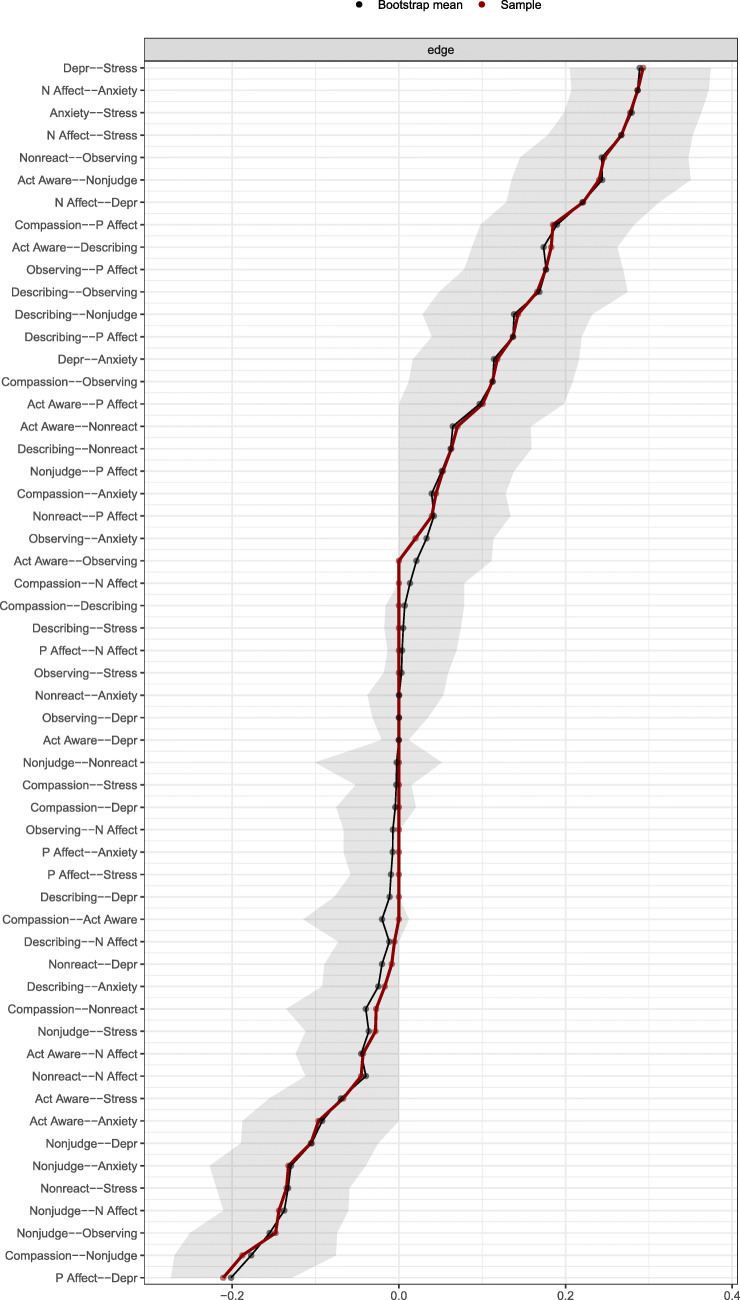
Sample mean, bootstrapped mean, and bootstrap-based confidence interval (gray area) for each edge

Accuracy checks of network parameters using bootstrapping and case-dropping indicated high accuracy. The CS coefficient for the order of strength centrality (i.e., the degree to which each node is connected to other nodes regardless of node community/cluster) in the full network was 0.60 indicating high robustness of centrality estimates. The CS coefficient for order of bridge strength centrality was 0.67 indicating that the bridge centrality estimates were very robust. Figure 3 shows 95% confidence intervals computed around each edge. We specifically inspected confidence intervals for edges linking protective and maladjustment nodes, as this was the major focus of the study. Overall, confidence intervals indicated that the edges linking protective and maladaptive nodes had a high degree of accuracy.

## Discussion

In the current study, we examined how facets of mindfulness, compassion, positive and negative affect, depression, and anxiety are interrelated, applying a network analytical approach. Consistent with the literature (Denollet and De Vries 2006), our network analysis demonstrated that both depression and anxiety were strongly and directly linked to stress and negative affect, but only depression was inversely related to positive affect. Positive affect, in turn, was associated with compassion, non-reactivity, mindful observing, and describing facets of mindfulness, which were not directly associated with depression. A non-judgmental attitude had a unique role in bridging protective and maladaptive factors and was inversely related to all aspects of emotional distress and negative affect. Non-reactivity and acting with awareness also acted as important bridge nodes between protective and maladaptive factors.

Regarding compassion, despite its important role in various fields of human activity, from health (Cochrane et al. 2019) to education (Al-Ghabban 2018) and business (Shuck et al. 2019), very little research to date has investigated its role in emotional well-being and as a potential protective factor against psychopathology. Compassion is oriented to improve the welfare of other human beings and entails an attitude toward others focused on understanding, caring, and helping them, particularly when they are perceived to be suffering and to be in need (Sprecher and Fehr 2005). Our study provides evidence revealing the role of compassion in the context of maladjustment and adjustment dimensions: The strong positive association between compassion and positive affect highlights the importance of compassion, suggesting a need to observe and describe the world through the lens of compassion to enhance positive affect and potentially alleviating or preventing symptoms of depression. It is well-established that negative emotions curb people’s thought–action repertoires, since they promote specific behaviors, such as attacking or fleeing, while positive emotions broaden people’s thought–action repertoires, promoting a broader range of thoughts and actions and help them to better adapt to stress and to recover from stressful experiences (Fredrickson et al. 2000). Positive emotions can constitute ongoing personal resources to draw from when confronted with unavoidable stressful events in later times, such as experiences of loss, typically associated to depression and negative emotions, or of a threat, typically associated to stress and anxiety. Along these lines, interestingly, in a recent study, patients with depression who were offered either traditional CBT or positive cognitive behavioral therapy (i.e., CBT focused on positive emotions and strengths) found positive CBT more pleasant and motivating, showing that paying explicit attention to positive affect in psychotherapy is rewarding, and can help to better treat depression (Geschwind et al. 2020). The strong and positive association between positive affect and compassion in our findings shows that a compassionate attitude toward our fellow human beings may help us to experience more emotions that are positive and constitute a buffer against depression.

Interestingly, our findings show that compassion is negatively related to non-judgment. This inverse association highlights a complex interplay between the two constructs. At this stage, any attempt to explain this relationship needs to rely on speculation about the generalizability of attitudes directed to oneself and toward others. To what extent does a non-judgmental stance toward one’s internal state imply that one also carries this attitude forward when considering the behavior and circumstances of others? Especially in individuals with high moral standards, the tendency to evaluate behavior and circumstances might motivate compassionate action leading to a prosocial expression of judgment. More research is necessary to investigate this possibility, but it has been reported that individuals with more self-compassion appear to have harsher criteria for their own moral behaviors (Wang et al. 2017). Additionally, in individuals with high proneness to guilt, and thus with high self-judgment, high levels of compassion for others could be expected. Generally, emotions imply action tendencies, which in the case of guilt are toward making atonement through good deeds. Indeed, guilt-prone individuals who harshly judge themselves are other-oriented, more capable of perspective taking, more likely to cooperate, share, and make sacrifices for others (Yang et al. 2010). This is also consistent with our finding that there is a small positive association between anxiety and compassion to others.

Another explanation might be that individuals who are judgmental of themselves experience higher levels of negative affect, which may also induce feelings of cognitive dissonance when being confronted with pressurizing or uncomfortable situations. When facing the suffering of others, one might be judgmental and feel uncomfortable of one’s own possibly more privileged situation, creating a state of tension and dissonance. Showing compassion and offering help might be used as a way to reduce negative affect and dissonance and enhance positive affect. Individuals who experience higher levels of cognitive dissonance for instance are more likely to donate for victims of natural disasters, and consequently are able to restore their mental state and feel better (Waters 2009). Regardless of an initial possibly egoistic motivation, it leads to change in behavior toward others, which in turn may influence attitudes, namely justifying one’s own behavior as the right one.

As the present study investigated trait mindfulness in a cross-sectional sample, non-judgmental awareness must not be interpreted in the intended meaning within MBIs or Buddhist practice. In the latter contexts, one could argue that judgments of behavior may naturally arise but are then deliberately suspended. Evaluation of other people’s behavior may then be considered in terms of skillful versus unskillful as opposed to good or bad (Feng et al. 2018). Such wise judgment that is based on insight and life experience might then be found to be highly associated with compassion for others. Future work in the context of MBI practice is therefore required.

Regarding the complex associations among all the variables involved in this cross-sectional network analysis, non-judgment had a unique role in bridging protective and maladaptive factors and was inversely related to all aspects of emotional distress and negative affect, a finding in line with the literature (Barcaccia et al. 2019; Tumminia et al. 2020). Non-reactivity and acting with awareness also acted as bridges between protective and maladaptive factors, showing how the mindful capacity of responding to both external and internal stressful events and acting with awareness, as opposed to reacting impulsively, represent an important set of skills when dealing with difficult situations, and may protect from symptoms of maladjustment. Positive affect, in turn, was associated with compassion, non-reactivity, observing, and describing facets of mindfulness, which were not directly associated with depression.

Non-judgment and observing were negatively related, a result also found by Siegling and Petrides (2016). This inverse association suggests that it is not enough to be capable of observing one’s inner states, but what is essential is learning to suspend judgment and accept them. Clinical observations suggest that depressed individuals are very introspective, i.e., can observe very thoroughly their inner states, but at the same time they struggle to accept them, to the contrary they often judge them very harshly. Non-judgment again emerges as one of the most important facets of mindful attitude in relation to well-being. Furthermore, observing and describing, as already evidenced by other studies (Baer et al. 2008; Medvedev et al. 2018), are the only two facets of mindfulness, which were not directly associated with psychopathology symptoms, except for a weak negative relation between describing and anxiety.

Our findings suggest that different aspects of mindfulness are important to inhibit negative affectivity and that a non-judging attitude may be particularly important. Using network analysis, this study illustrates the network of mindfulness, compassion, affect, and emotional distress and highlights the central role that a non-judging attitude may play alongside a non-reacting attitude and acting with awareness, as well as the pivotal role of compassion.

### Limitations and Future Research

This study presents some limitations. First, its cross-sectional design precludes conclusions regarding the causal direction among the explored variables. Future research should investigate these variables longitudinally and experimentally. Second, we used a community sample: future studies could investigate the same variables in clinical samples diagnosed with depressive or anxiety disorders. Third, we only used self-report measures that may introduce a response bias. However, psychometric properties of the FFMQ and DASS-21 were enhanced using ordinal to interval Rasch transformation that reduces measurement error enhance precision of assessment.

Our findings evidenced possible unique roles of non-judging and to some extent compassion in relation to negative affect and psychological distress, and these dimensions could be enhanced in prevention/intervention programs. Specifically, development of a revised comprehensive judgment scale is warranted with subscales focusing on adaptive and maladaptive forms of self-judgment including judgment of others, judgment of the environment, and perceived judgment of other people. To test the hypothesis that compassion training will be effective in treating symptoms of depression, a three-arm randomized controlled trial could be conducted comparing three different intervention protocols: (1) mindfulness-based intervention, (2) compassion-based intervention, and (3) cognitive-behavioral therapy as active control. Such a comparison could increase our understanding of the active and effective role of compassion and different aspects of judgment in treatments and shed light on which mechanisms effectively drive change. As such, our findings can promote the evaluation of mindfulness-based resilience programs that aim treat or prevent emotional disorders and enhance psychological well-being.
